# Awareness of Emergency Medical Care Among School Teachers in Rabigh City

**DOI:** 10.7759/cureus.99260

**Published:** 2025-12-15

**Authors:** Hamoud Abdullah A Alharbi, Mutlaq Marzouq Almutairi, Fayez Sameer Alharbi, Abdullah Muaybid Al Harbi, Meshari Murayshid Almuthaybiri, Bader Ali Alharbi, Khaled Hamdan Almoteri, Shuaa Abdullah Alharbi, Sahmi Ajmi Almotari, Abdullah Mohammed Alharbi, Surur Murayshid Almuthaybiri

**Affiliations:** 1 Paramedics, Saudi Red Crescent Authority, Qassim, SAU; 2 Paramedics, Primary Health Care Al Badai' Al Ulya, Ministry of Health, Qassim, SAU

**Keywords:** cardiopulmonary resuscitation, cross-sectional study, emergency preparedness, first aid knowledge, saudi arabia, schoolteachers

## Abstract

Background

School children are at risk of sudden injuries and illnesses at school, especially in rural areas where medical facilities are limited. Since teachers are often the first responders, their awareness of emergency medical care is crucial for timely and effective management. This study aimed to assess the level of knowledge of emergency medical care among school teachers in Rabigh Governorate, Saudi Arabia.

Methods

A cross-sectional study was conducted among 372 educators from both government and private institutions. Data were collected using a validated, self-administered questionnaire encompassing socio-demographic characteristics, prior training, emergency experiences, and item-specific first aid knowledge. Stata 17 (StataCorp, College Station, USA) was used for data analysis.

Results

Among 372 teachers, the mean first-aid knowledge score was 5.17 ± 1.90 (range 0-10). Overall, 97 (26.1%) had poor knowledge, 220 (59.1%) had average knowledge, and 55 (14.8%) had good knowledge. Highest item-level accuracy was seen for recognizing anaphylaxis/EpiPen™ (Viatris, Canonsburg, USA) use 247 (66.4%), washing dog-bite wounds 245 (65.9%), and external bleeding control 241 (64.8%). Lowest accuracy was for CPR 89 (23.9%), snake/insect bites 104 (27.9%), and burns 122 (32.8%). Prior first-aid training was the strongest predictor of good knowledge (AOR = 7.13; 95% CI: 4.21-13.25; p < 0.001). Other significant predictors were ≥15 years’ experience (AOR = 3.50; 95% CI: 0.95-5.80; p = 0.019), postgraduate education (AOR = 1.80; 95% CI: 0.97-3.60; p = 0.037), male gender (AOR = 2.20; 95% CI: 0.60-3.10; p = 0.037), government school employment (AOR = 1.44; 95% CI: 0.38-2.98; p = 0.047), teaching at secondary/higher level (AOR = 3.60; 95% CI: 0.95-5.90; p = 0.001), and prior management of student emergencies (AOR = 2.40; 95% CI: 1.30-4.10; p < 0.001).

Conclusion

School teachers in Rabigh exhibited moderate knowledge of first aid, with notable deficiencies in crucial life-saving skills such as cardiopulmonary resuscitation (CPR) and seizure management. Factors such as training, advanced education, substantial teaching experience, and prior exposure to emergencies were identified as strong predictors of competency. These results highlight the urgent need to implement mandatory, standardized first aid training and refresher courses for all educators, with a special emphasis on primary-level teachers and critical emergencies.

## Introduction

Emergency medical care, commonly referred to as first aid, involves the immediate assistance provided to individuals experiencing acute illness or injury before the arrival of professional medical personnel [1]. In many emergencies, bystanders at the scene offer this initial care, acting as intermediaries until emergency services arrive [2]. First aid is essential for stabilizing a victim’s condition, regardless of whether the situation is minor or life-threatening. Its primary objectives are to preserve life, prevent deterioration, and promote recovery while awaiting advanced medical care [3].

School-aged children spend a significant portion of their day taking part in educational activities, during which time their safety is a primary concern. A considerable proportion of childhood injuries occur within the school environment; it is estimated that between 10% and 25% of childhood injuries take place during school hours or on school premises [4]. Children are particularly susceptible to injuries due to physical and developmental factors. Their limited awareness of risks often prevents them from recognizing potential dangers, while their smaller size and more delicate physiology further heighten their vulnerability [4, 5]. Given this combination of frequent exposure and increased risk, the prompt delivery of first aid within schools is essential. Timely and appropriate first aid can significantly reduce harm and, in many cases, save lives.

In school emergencies, teachers often act as first responders. In the absence of on-site medical personnel, teachers and other staff members are responsible for providing initial care until emergency professionals arrive [6, 7]. Thus, beyond teaching, teachers are responsible for students’ safety and well-being, with their first aid skills often directly influencing emergency outcomes. This means that prompt action immediately after an injury or collapse is crucial [8, 9]. Teachers with strong first aid skills can prevent complications, reduce injury severity, and sometimes avert death. Conversely, a lack of first aid competence may lead to preventable worsening of a student’s condition. Therefore, teachers should be prepared to act quickly and confidently in emergencies. However, evidence shows many are not sufficiently trained to do so.

Studies in major Saudi Arabian cities reveal significant gaps in teachers’ first aid knowledge and preparedness [3, 10]. A survey in Riyadh found that only around 14.9% of teachers had good first aid knowledge [4], with most possessing limited knowledge of basic practices. Research in Jeddah also revealed gaps in teachers’ practical readiness. All teachers had heard of first aid or received some information about it, yet only 4.5% felt confident in administering it [4]. More than half did not feel confident at all. Together, these findings reveal a concerning knowledge and training gap among urban teachers, which has prompted calls for better first aid training in cities. However, there is limited information about teachers in rural areas. The situation in rural communities could be just as serious - or worse - than in cities, since they often have less immediate access to emergency care.

In rural areas, access to advanced medical facilities and emergency services is often limited compared to cities [11]. Ambulance response times are longer, and healthcare resources are scarcer [12]. Consequently, schools in rural areas may experience greater delays in receiving professional care for injured children. Rabigh Governorate, located in western Saudi Arabia, is one such area. It comprises Rabigh City and several surrounding towns, encompassing over 30 schools and more than 30,000 students. Approximately 1,000 teachers work at these schools. Given that emergency services may be delayed, the response of school staff in Rabigh is crucial. Yet, little is known about the level of teachers’ emergency preparedness in this region. Published data on teachers’ first aid knowledge in Saudi Arabia, especially in rural schools, is limited. Therefore, assessing first aid knowledge in these areas is essential.

This cross-sectional study aims to evaluate first aid knowledge among schoolteachers in Rabigh Governorate. Specifically, we will measure teachers’ understanding and confidence in basic first aid, with the goal of identifying and addressing any critical gaps and training needs. Insights from the study can guide targeted training and emergency policies among Rabigh schools. Furthermore, the findings may also benefit other rural areas in Saudi Arabia and similar regions globally. Enhancing teachers’ first aid competencies ultimately contributes to safer school environments and improved student health outcomes.

Research objectives

Primary Objective: To assess the level of knowledge and competency in emergency care among schoolteachers in a rural area, specifically, to evaluate whether teachers can recognize student emergencies and respond with appropriate first aid.

Secondary Objective: To use the study results to form recommendations for improving first aid preparedness through programs or guidelines if knowledge gaps are found. This aims to strengthen teachers’ capacity to manage emergencies in Rabigh and similar rural areas.

## Materials and methods

Study design and duration

A cross-sectional observational study was conducted to assess teachers’ awareness of emergency care and first aid. The research took place in Rabigh City and its surrounding towns within Rabigh Governorate, Saudi Arabia, from July 2024 to June 2025. The target population consisted of all teachers, regardless of gender, employed in public and private schools at all educational levels: primary, intermediate, and secondary. Approximately 30 schools in Rabigh and its nearby villages were included. Participants were required to have at least one year of teaching experience and to be actively engaged in teaching during the study period. Non-teaching staff, including administrators, nurses, and janitors, were excluded from the study.

After obtaining necessary permissions, eligible teachers were invited to participate through their school administration. Convenience sampling was employed to enhance participation rates, and responses were voluntary. The importance of the research and its contribution to student safety were emphasized to potential participants.

The minimum sample size was determined using a standardized formula for cross-sectional surveys. Assuming a 50% prevalence of baseline knowledge, a 95% confidence interval, and a 5% margin of error, the calculated sample size was approximately 300 participants, based on an estimated total population of roughly 1,000 teachers in Rabigh Governorate. This calculation was performed using the Raosoft sample size calculator (Raosoft Inc., Seattle, USA). To account for potential non-response or incomplete surveys, a minimum of 400 complete responses was targeted. Ultimately, a response rate of 93% was achieved, resulting in a final sample of 372 participants.

A structured electronic questionnaire was used for data collection. It was initially developed in English, translated into Arabic, and then back-translated to ensure accuracy and linguistic consistency. The questionnaire comprised three sections: demographics and background information, first aid knowledge (based on scenarios), and an unspecified third section. Two emergency medicine physicians reviewed the questionnaire to confirm content validity and ensure relevance to the study objectives. A pilot test was subsequently conducted with ten local educators (who were excluded from the main study) to evaluate clarity and timing. Based on feedback from the pilot, minor revisions were made, including the simplification of medical terminology and the addition of pictorial cues for specific questions. The finalized questionnaire was distributed online via platforms such as Google Forms or Qualtrics (Qualtrics LLC, Provo, USA), with access provided through a secure link or QR code. 

First-aid knowledge was assessed using 10 scenario-based, single-best-answer items covering common school emergencies (e.g., anaphylaxis/EpiPen (Viatris, Canonsburg, USA) use, choking, external bleeding, burns, seizures, epistaxis, heat illness, asthma exacerbation, suspected fractures, and animal/insect bites). Each item was dichotomously scored: a correct response = 1 point; an incorrect response or “I don’t know” = 0 points. All items were equally weighted, and no partial credit or negative marking was applied. For each participant, item scores were summed to a total knowledge score ranging from 0 to 10 (higher scores indicate greater knowledge). Consistent with a priori thresholds, total scores were interpreted as: Poor knowledge: < 5, Average knowledge: 5-7, Good knowledge: ≥ 8

After obtaining ethical approval and coordinating with the education authorities, we contacted the relevant school administrations. Principals were asked for permission to invite their teachers to participate. Once permission was granted, teachers received a survey link along with detailed study information. They were then assured of their anonymity and confidentiality and informed that participation was voluntary. They were permitted to complete the questionnaire at their convenience within a period of 3 to 4 weeks, during which periodic reminders were sent to enhance response rates. The anonymous, self-administered format was designed to encourage honest responses.

Informed consent was obtained at the beginning of the survey, with the initial page providing an introduction to the study and a checkbox for teachers to confirm their consent after reviewing the information. No personal identifiers such as names, ID numbers, or school names were collected. Each response was automatically assigned a unique identifier by the survey system.

During the data collection phase, our team carefully monitored response rates. When low participation was observed in certain schools or groups, such as female teachers in rural areas, we implemented targeted follow-up measures to address the issue. These included additional reminders, outreach efforts, direct communication through liaison teachers, and school visits.

Data analysis

Data were analyzed using Stata version 17 (StataCorp, College Station, USA). Descriptive statistics were reported as frequencies and percentages for categorical variables. First aid knowledge scores were labeled as poor, average, or good. Group differences were assessed using the chi-square test. To identify predictors of good first aid knowledge (score ≥ 8), we performed multivariable logistic regression analysis. A p-value of less than 0.05 was considered significant.

## Results

A total of 372 teachers participated in the study, including 194 (52.2%) males and 178 (47.8%) females. Participants’ ages ranged from 21 to over 50 years: specifically, 40 (10.8%) were aged 21-30 years, 93 (25.0%) were 31-40 years, 188 (50.5%) were 41-50 years, and 51 (13.7%) were above 50 years. The majority, 296 (76.9%), were Saudi nationals, while 86 (23.1%) were non-Saudi. Most participants, 318 (85.5%), held a bachelor’s degree, whereas 23 (6.2%) had a diploma, 25 (6.7%) a master’s degree, and six (1.6%) a Ph.D. (Table 1).

**Table 1:** Participant characteristics (N = 372)

Characteristic	n (%)
Sex	
Male	194 (52.2%)
Female	178 (47.8%)
Age, years	
21–30 years	40 (10.8%)
31–40 years	93 (25.0%)
41–50 years	188 (50.5%)
≥50 years	51 (13.7%)
Nationality	
Saudi	296 (76.9%)
Non-Saudi	86 (23.1%)
Education Level	
Diploma	23 (6.2%)
Bachelor	318 (85.5%)
Master	25 (6.7%)
Ph.D.	6 (1.6%)
Years of teaching	
<5 years	54 (14.5%)
5–10 years	52 (14.0%)
11–15 years	106 (28.5%)
≥15 years	160 (43.0%)
School level	
Elementary	138 (37.1%)
Medium	118 (31.7%)
High	116 (31.2%)
School type	
Governmental	265 (71.2%)
Private	107 (28.8%)
Section	
Boys	194 (52.2%)
Girls	178 (47.8%)
Prior first aid training	232 (62.4%)
Ever managed a student emergency	180 (48.4%)

In terms of teaching experience, 54 (14.5%) had less than five years of experience, while 52 (14.0%) had 5-10 years, 106 (28.5%) had 11-15 years, and 160 (43.0%) had 15 years or more. In terms of school levels, participants were distributed as follows: 138 (37.1%) at the elementary level, 118 (31.7%) at the intermediate level, and 116 (31.2%) at the high school level. With respect to school type, 265 (71.2%) were employed in government schools, while 107 (28.8%) worked in private institutions. Notably, 194 (52.2%) participants were taught in boys’ sections and 178 (47.8%) in girls’ sections. A total of 232 (62.4%) reported having received prior first aid training, while 180 (48.4%) indicated that they had previously managed a student emergency.

Among the 372 participants evaluated for their knowledge of correct first aid responses, accuracy varied depending on the emergency scenario being discussed. The highest accuracy was for anaphylaxis recognition, where 247 participants (66.39%) correctly identified the need for EpiPen administration. For dog bite wound care, 245 participants (65.86%) identified washing the wound as the correct response. Techniques for controlling external bleeding, such as applying direct pressure and elevating the limb, were correctly recognized by 241 participants (64.78%).

Beyond these areas of relative strength, participant performance showed notable variability across other emergency scenarios. For example, in epistaxis management, 201 participants (54.03%) identified the correct practice, which involves leaning forward and applying nasal pressure. In choking incidents, 178 (47.84%) recognized that the appropriate response involves performing abdominal thrusts and avoiding water intake or back slaps in cases of severe obstruction. Similarly, 174 (46.77%) correctly indicated that a child should be wrapped in cloth during fire-related emergencies. Knowledge of seizure management was slightly lower, with 155 participants (41.6%) identifying appropriate interventions, including protecting the affected individual from injury, placing them in the recovery position, and avoiding restraints or inserting objects into the mouth. The same number of participants (155; 41.66%) addressed heat-related illness correctly, demonstrating an ability to differentiate between exhaustion and heat stroke, and to implement cooling measures or activate Emergency Medical Services (EMS)as needed. Moreover, 152 participants (40.86%) accurately identified how to manage asthma exacerbations, including positioning the patient upright and administering a short-acting beta-agonist (SABA) via inhaler or spacer.

In contrast, fewer participants correctly answered questions on suspected fractures (135; 36.29%), burns (122; 32.79%), and snake or insect bites (104; 27.95%), while CPR-related knowledge had the lowest accuracy, with only 89 participants (23.92%) providing correct responses covering compression rate, depth, and activation sequence (Table 2).

**Table 2:** Item-level knowledge performance (N = 372) EMS: Emergency Medical Services; SABA: short-acting beta-agonist EpiPen™ is a brand owned by Viatris, Canonsburg, USA.

Knowledge item (correct action/recognition)	Correct, n (%)
Choking management (abdominal thrusts; avoid water/back-slaps in severe airway obstruction)	178 (47.84%)
Seizure first aid (protect from injury, recovery position, no restraints/objects in mouth)	155 (41.6%)
External bleeding control (direct pressure, elevation)	241 (64.78%)
Suspected fracture (immobilize, avoid moving limb)	135 (36.29%)
Epistaxis (forward lean, nasal pressure; avoid head tilt back)	201 (54.03%)
Burns (cool running water ≥15 min; avoid ice/ointment initially)	122 (32.79%)
Asthma exacerbation (sit upright, allow SABA inhaler/spacer)	152 (40.86%)
Anaphylaxis recognition & EpiPen use (rapid administration)	247 (66.39%)
Heat illness (differentiate exhaustion vs stroke; cooling/EMS activation as appropriate)	155 (41.66%)
CPR knowledge (adult compression rate/depth, activation sequence)	89 (23.2%)
Snake/insect bite management	104 (27.95%)
Should the site dog bite be washed (yes)	245 (65.86%)
Fire management (wrap the child in cloth)	174 (46.77%)

The assessment of first aid knowledge among the 372 participants indicated a moderate level of understanding. The mean knowledge score was 5.17 with a standard deviation of 1.90, and scores ranged from 0 to 10. Based on performance categories, 97 participants (26.07%) demonstrated poor knowledge (scores < 5), while the majority, 220 participants (59.1%), achieved an average level of knowledge (scores 5-7). Only 55 participants (14.8%) attained a good level of knowledge, defined as a score of ≥ 8. These results highlight the need for targeted educational interventions. Table 3.

**Table 3:** First aid knowledge scores and levels among participants

Knowledge score (out of 10)	Value
Mean ± SD (Range)	5.17 ± 1.90 (0–10)
Poor knowledge (score < 5) n (%)	97 (26.07%)
Average knowledge (score 5–7) n (%)	220 (59.13%)
Good knowledge (score ≥ 8) n (%)	55 (14.78%)

Among the 372 participants, previous first aid training was significantly associated with higher knowledge scores (p < 0.001). Only 9.9% of those who had received training demonstrated poor knowledge, compared to 52.9% of those without training. Teaching experience was also significant (p = 0.0178); teachers with 5-10 years of experience most often had average scores (69.6%), while those with 15 years or more were the most likely to possess good knowledge (19.0%). Education level also mattered (p < 0.001): 54.5% of diploma holders had poor knowledge, whereas 29.1% of postgraduates demonstrated good knowledge. Nationality did not show a significant difference (p = 0.2468). School type made a difference (p = 0.0022), with private school teachers demonstrating greater knowledge (23.1%) compared to government school teachers (10.1%). There were also differences by teaching section (p = 0.007), with secondary and higher education teachers scoring better (27.4%) than primary teachers (10.0%). Lastly, teachers who had previously managed student emergencies also had greater knowledge (21.5% good versus 9.8% without such experience, p = 0.003) (Table 4).

**Table 4:** First aid knowledge scores, levels, subgroup comparisons, and p-values (N = 372) ^#^chi-square test

Variables	Poor n (%) n=97	Average n (%) n=220	Good n (%) n=55	p-value^#^
Prior first aid training				
Yes	23 (9.9%)	175 (75.4%)	34 (14.7%)	<0.001
No	74 (52.9%)	45 (32.1%)	21 (15.0%)	
Years of teaching				
<5 years	27 (32.5%)	44 (53.0%)	12 (14.5%)	0.0178
5–10 years	24 (20.9%)	80 (69.6%)	11 (9.6%)	
11–15 years	26 (27.4%)	52 (54.7%)	17 (17.9%)	
≥15 years	20 (25.3%)	44 (55.7%)	15 (19.0%)	
By education level				
Diploma	18 (54.5%)	13 (39.4%)	2 (6.1%)	<0.001
Bachelor’s	71 (27.3%)	159 (61.2%)	30 (11.5%)	
Postgraduate	8 (10.1%)	48 (60.8%)	23 (29.1%)	
By nationality				
Saudi	77 (24.1%)	193 (60.3%)	50 (15.6%)	0.2468
Non-Saudi	20 (38.5%)	27 (51.9%)	5 (9.6%)	
By school type				
Government	74 (31.1%)	140 (58.8%)	24 (10.1%)	0.0022
Private	23 (17.2%)	80 (59.7%)	31 (23.1%)	
By section taught				
Primary	53 (35.3%)	82 (54.7%)	15 (10.0%)	0.007
Middle	28 (20.3%)	93 (67.4%)	17 (12.3%)	
Secondary/Higher	16 (19.0%)	45 (53.6%)	23 (27.4%)	
Ever managed a student emergency?				
Yes	32 (20.3%)	92 (58.2%)	34 (21.5%)	0.003
No	65 (30.4%)	128 (59.8%)	21 (9.8%)	

Multivariable logistic regression analysis identified several independent predictors of proficient first aid knowledge (score ≥ 8). Prior first aid training was the most significant factor, with educators who had received training having an adjusted odds ratio (AOR) of 7.13 (95% CI: 4.21-13.25, p < 0.001) for substantial knowledge compared to those without training. Educators with more than 15 years of experience had an AOR of 3.50 (95% CI: 0.95-5.80, p = 0.019) for adequate knowledge. Fewer years of experience did not show a significant correlation.

Beyond teaching experience, educational attainment demonstrated a graded relationship with knowledge. Postgraduate degree holders (AOR 1.80, 95% CI 0.97-3.60, p = 0.037) and doctoral degree holders (AOR 3.40, 95% CI 1.21-6.10, p = 0.082) exhibited greater odds of possessing adequate knowledge compared to individuals with a bachelor’s degree. For doctoral degree holders, the p-value (0.082) indicated marginal statistical significance, as it slightly exceeded the conventional threshold for significance (p < 0.05); thus, this result should be interpreted with caution. In addition, male educators showed higher odds than their female counterparts (AOR 2.20, 95% CI 0.60-3.10, p = 0.037). The type of school was also a significant factor. Teachers in government institutions were more likely to possess sound knowledge than those in private schools (AOR 1.44, 95% CI 0.38-2.98, p = 0.047).

Moreover, teaching level and previous experience in emergency management were both significant predictors. Educators at secondary or higher levels had much higher odds of possessing good knowledge than primary-level teachers (AOR 3.60, 95% CI 0.95-5.90, p = 0.001), while teachers who had managed student emergencies were more than twice as likely to achieve a high score (AOR 2.40, 95% CI 1.30-4.10, p < 0.001). Nationality showed no significant link (p = 0.921) (Table 5).

**Table 5:** Multivariable logistic regression of factors associated with good first aid knowledge (score ≥ 8), (n=372)

Predictor	Adjusted OR (95% CI)	p-value
Prior first aid training		
No (reference)		
Yes	7.13 (4.21–13.25)	<0.001
Years of teaching		
0–5 years (reference)		
6–10 years	0.52 (0.06–1.52)	0.892
11–15 years	1.60 (0.80–2.80)	0.40
>15 years	3.50 (0.95–5.80)	0.019
Education		
Bachelor’s (reference)		
Diploma/Certificate	0.78 (0.34–2.67)	0.721
Postgraduate	1.80 (0.97–3.60)	0.037
Ph.D.	3.40 (1.21–6.10)	0.082
Gender		
Female (reference)		
Male	2.20 (0.60–3.10)	0.037
Nationality		
Non-Saudi (reference)		
Local	1.02 (0.30–2.25)	0.921
School type		
Private (reference)		
Government	1.44 (0.38–2.98)	0.047
Section taught		
Primary (reference)		
Middle	0.80 (0.54–2.11)	0.712
Secondary/Higher	3.60 (0.95–5.90)	0.001
Ever managed a student emergency?		
No (reference)		
Yes	2.40 (1.30–4.10)	<0.001

## Discussion

Our investigation revealed that schoolteachers’ overall knowledge of first aid was moderate. The average score was slightly above 5 out of 10, with approximately 15% of participants attaining a “good” level of knowledge (score ≥8). This result corroborates earlier studies conducted in Saudi Arabia. For instance, a 2019 survey in Riyadh found that only 14.9% of male educators achieved a satisfactory score in first aid knowledge, defined as a score of 60% or higher on their assessment [3]. Similarly, an earlier study conducted in Khamis Mushayt reported that only 19.6% of secondary school teachers possessed knowledge of first aid [13]. These findings collectively highlight a significant deficiency in first aid preparedness among educators. Furthermore, a recent systematic review confirmed that the majority of Saudi schoolteachers lack sufficient knowledge in managing health emergencies, emphasizing the need for improvement [14].

In our evaluation of specific first aid scenarios, we observed significant variations in the knowledge of teachers. Participants excelled in questions related to recognizing anaphylaxis and using an epinephrine injector, with approximately two-thirds answering correctly. Comparable levels of accuracy were also observed for basic wound care (e.g., treating a dog bite) and hemorrhage control, with approximately 65% of the participants answering correctly. They also demonstrated a moderate level of understanding regarding the management of nosebleeds, with 54% knowing that the child should lean forward and apply pressure to the nose. These strengths are consistent with findings from other regions. For example, a study conducted in Eastern Saudi Arabia reported that 55.3% of teachers were knowledgeable about the proper technique for abdominal thrusts in cases of choking [15], which is comparable to the 48% within our cohort who accurately responded to the choking scenario. However, essential life-saving skills were deficient. Only 23.9% of our participants were familiar with the fundamental procedures of cardiopulmonary resuscitation (CPR). Such a deficiency is unsurprising, as previous surveys have reported limited CPR proficiency among educators in the absence of specialized training [13, 15]. Our investigation also revealed limited awareness in specific domains, such as severe burns (approximately 33% answered correctly) and snakebite first aid (approximately 28% correct). Furthermore, a separate study conducted in Saudi Arabia found that only 28.6% of teachers were knowledgeable about the appropriate management of chemical eye burns (rinsing with water), highlighting comparable knowledge deficiencies in regard to common medical emergencies [16].

Among all predictors examined, prior first aid training had the most significant influence on knowledge levels. Teachers who had received formal training performed far better on the knowledge assessment than those without training (p < 0.001). In fact, only around 10% of previously trained teachers scored in the “poor” knowledge range, compared to over 50% of untrained teachers. The literature strongly supports this result. Multiple studies have found that having formal first aid training is the strongest predictor of a teacher’s emergency knowledge and confidence [17, 18].

A higher level of academic education was another significant factor associated with improved first aid knowledge in our study. For example, an Ethiopian study demonstrated that teachers with higher educational attainment exhibited greater expertise and accuracy in emergency response [17].

Our findings indicate that teaching experience contributes to first aid knowledge in a nuanced way. Educators with the most extensive tenure (≥15 years) demonstrated higher odds of possessing good knowledge scores (AOR 3.5, p = 0.019) compared to those with fewer years of experience. Conversely, some studies report no significant enhancement in first aid knowledge solely attributable to the number of years in service [16].

Male educators demonstrated significantly higher average scores in first aid knowledge compared to female educators. Additionally, a comparable study conducted in Saudi Arabia noted that male teachers, especially those under 40 years of age, achieved higher scores in emergency knowledge than their female counterparts [16]. Potential explanations for this may include disparities in access to training, as, historically, male staff or coaches might have had greater opportunities to attend first aid courses, or differences in self-education. However, it is important to note that not all research supports this pattern. For example, an extensive study of public school teachers in Qassim concluded that there was no significant difference in knowledge between male and female teachers once other variables were controlled [15].

Our findings also revealed that school environment and professional experience markedly influenced first aid knowledge. Teachers in private schools initially achieved higher scores; however, following adjustment, educators in government schools exhibited a greater likelihood of possessing adequate knowledge, likely attributable to confounding factors such as seniority and qualifications [4]. Secondary and high school teachers were more knowledgeable than primary teachers, reflecting greater exposure to health-related topics and student emergencies [4]. Previous experience in emergency management was associated with enhanced knowledge levels (21.5% compared to 9.8%, p = 0.003), underscoring the importance of practical exposure and simulation exercises. Conversely, years of service alone did not guarantee proficiency [13, 19].

This study has several limitations that should be taken into account when interpreting the findings. This is a cross-sectional, self-reported study that cannot establish causality and may be affected by recall or social desirability bias. The questionnaire-based design assessed theoretical knowledge rather than practical performance, which may have led to an overestimation of actual skill levels. Moreover, the study was limited to one governorate, which may reduce its generalizability, while unmeasured factors, such as school policies or access to nurses, could have influenced the results.

## Conclusions

This study found that school teachers in Rabigh possess a moderate level of first aid knowledge, with notable gaps reported in critical skills, including CPR, seizure management, and burn care. Factors such as prior training, extensive teaching experience, postgraduate qualifications, male gender, working in government schools, teaching at the secondary or higher educational levels, and prior exposure to student emergencies serve as significant predictors of enhanced knowledge. This underscores the critical need for the implementation of compulsory, standardized first aid training and periodic refresher courses for all teaching personnel, with particular emphasis on primary education instructors who demonstrate lower levels of preparedness. Enhancing teachers’ first aid capabilities through structured training programs and simulation exercises, supported by appropriate policy initiatives, will increase their confidence and capacity to respond swiftly and effectively in the event of school emergencies, thereby safeguarding students’ safety and well-being.

## Appendices

Teacher First Aid Knowledge - Study Questionnaire (Rabigh, 2024-2025)

Instructions

• This survey is anonymous and voluntary. Do not include your name or school name. • Choose the single best answer for each knowledge question. Select “I don’t know” if unsure. • Estimated time: 8-10 minutes.

Section A. Informed Consent

Purpose: To assess teachers’ awareness of emergency care and first aid in schools. Risks/Benefits: Minimal risk; potential benefit to student safety through improved understanding. Confidentiality: No personal identifiers are collected. Data are reported in aggregate.

1. Consent ☐ I have read the information above and agree to participate.

Section B. Demographics and Background

Please choose one option for each item.

Sex ☐ Male ☐ Female ☐ Prefer not to say

Age group (years) ☐ 21-30 ☐ 31-40 ☐ 41-50 ☐ ≥50

Nationality ☐ Saudi ☐ Non‑Saudi

Highest education level ☐ Diploma/Certificate ☐ Bachelor’s ☐ Master’s ☐ PhD

Years of teaching experience ☐ <5 ☐ 5-10 ☐ 11-15 ☐ ≥15

School level (primary assignment) ☐ Elementary ☐ Intermediate ☐ Secondary/High

School type ☐ Government ☐ Private

Section taught ☐ Boys ☐ Girls ☐ Both

Have you ever received formal first‑aid training? ☐ Yes (year: _____) ☐ No

Have you ever managed a student emergency at school? ☐ Yes ☐ No

Section C. First Aid Knowledge (Single Best Answer)

Select one answer per question.

Core Items (used for the 10‑point knowledge score)

C1. Anaphylaxis: A student with sudden facial swelling, hives, wheeze, and dizziness after eating peanuts. What is the first action? A. Give water and observe B. Lay flat and wait for EMS C. Administer epinephrine auto‑injector (EpiPen) immediately D. Give oral antihistamine E. I don’t know

C2. Choking (severe airway obstruction, conscious adolescent): What should you do? A. Offer water B. Back slaps only C. Abdominal thrusts (Heimlich maneuver) D. Ask the student to lie down E. I don’t know

C3. External bleeding (forearm laceration): Best immediate measure? A. Rinse with hydrogen peroxide B. Apply direct pressure; elevate limb C. Apply tourniquet first D. Remove any embedded object E. I don’t know

C4. Burns (hot liquid on forearm, intact skin): Initial first aid? A. Apply ice directly B. Apply ointment/butter C. Cool under running water ≥15 minutes D. Pop blisters E. I don’t know

C5. Seizure (generalized, stopped after 1 min): Appropriate action now? A. Force spoon into mouth B. Protect from injury; place in recovery position; monitor breathing C. Restrain arms and legs D. Give water immediately E. I don’t know

C6. Epistaxis (nosebleed): Correct position and technique? A. Sit leaning forward; pinch soft part of the nose B. Tilt head back; insert tissue C. Lie flat; cold pack on neck D. Breathe through mouth only E. I don’t know

C7. Heat illness (dizzy athlete after PE on hot day): Best immediate step? A. Keep in sun and observe B. Move to shade/cool area; start active cooling; hydrate if conscious C. Give salt tablets D. Ignore if temperature <39°C E. I don’t know

C8. Asthma exacerbation (wheeze, can speak in short phrases): First aid? A. Lie the student flat B. Sit upright; give SABA inhaler (with spacer if available); assess response C. Offer antihistamine D. Ask to run to nurse’s office E. I don’t know

C9. Suspected fracture (deformity after fall): What should you do before transport? A. Attempt to straighten the limb B. Apply hot pack C. Immobilize/splint in the found position; avoid movement D. Massage area E. I don’t know

C10. Bites/Stings (snake or insect, swelling and pain): Best initial approach? A. Cut and suck the wound B. Apply tourniquet tightly C. Keep limb at heart level; remove constrictive items; seek urgent care D. Apply ice directly for 30 minutes E. I don’t know

Optional Extension Items (for item‑level analysis; not required for 10‑point score)

E1. Dog bite (shallow skin break): First step? A. Cover immediately without washing B. Wash thoroughly with soap and running water C. Apply powder/antibiotic and bandage D. Ignore if small E. I don’t know

E2. Fire/Flame on clothing: Best immediate action for a child whose shirt ignites? A. Run to create airflow B. Stop, drop, and roll; then cover with cloth/blanket C. Pour oil on the flames D. Remove clothing while running E. I don’t know

E3. CPR (unresponsive, not breathing normally): What is the correct first sequence? A. Call EMS/activate help; start chest compressions at 100-120/min, ~5-6 cm depth B. Give two breaths first C. Check pulse for 60 seconds D. Place in recovery position and wait E. I don’t know

Section D. (Optional) Confidence and Training Needs

Rate your agreement with each statement. 1 = Strongly disagree … 5 = Strongly agree.

* I feel confident managing common school emergencies.

* I know when to activate EMS for student emergencies.

* I would attend school‑based first‑aid refresher training if offered.

* Our school has adequate first‑aid supplies and an emergency plan.

Response options: 1 ☐ 2 ☐ 3 ☐ 4 ☐ 5 ☐

Scoring Key and Interpretation (for researchers)

Core knowledge score (0-10):

- Award 1 point for each correct response to C1-C10; 0 points for incorrect/“I don’t know”.

- Total = sum(C1…C10), range 0-10.

- Interpretation: Poor <5; Average 5-7; Good ≥8.

- Unanswered items are scored as incorrect.

*Item‑level performance:*

- Report n (%) correct for each item C1-C10 and, if included, E1-E3.

- Logistic regression outcome (as used in the manuscript):

- Define “good knowledge” as score ≥8 on the 10‑item core scale.

Thank you for your participation!
